# How water-mediated hydrogen bonds affect chlorophyll *a*/*b* selectivity in Water-Soluble Chlorophyll Protein

**DOI:** 10.1038/s41598-019-54520-4

**Published:** 2019-12-03

**Authors:** Alessandro Agostini, Elena Meneghin, Lucas Gewehr, Danilo Pedron, Daniel M. Palm, Donatella Carbonera, Harald Paulsen, Elmar Jaenicke, Elisabetta Collini

**Affiliations:** 1 Department of Chemical Sciences, University of Padova, via Marzolo 1, 35131 Padova, Italy; 2 Institute of Molecular Physiology, Johannes Gutenberg-University, Johannes-von-Müller-Weg 6, 55128 Mainz, Germany; 3 Institute of Molecular Physiology, Johannes Gutenberg-University, Jakob-Welder-Weg 26, 55128 Mainz, Germany

**Keywords:** Biophysical chemistry, Chemical biology, X-ray crystallography, Biophysical chemistry, Photosynthesis, Raman spectroscopy, Biochemistry

## Abstract

The Water-Soluble Chlorophyll Protein (WSCP) of *Brassicaceae* is a remarkably stable tetrapyrrole-binding protein that, by virtue of its simple design, is an exceptional model to investigate the interactions taking place between pigments and their protein scaffold and how they affect the photophysical properties and the functionality of the complexes. We investigated variants of WSCP from *Lepidium virginicum* (Lv) and *Brassica oleracea* (Bo), reconstituted with Chlorophyll (Chl) *b*, to determine the mechanisms by which the different Chl binding sites control their Chl *a*/*b* specificities. A combined Raman and crystallographic investigation has been employed, aimed to characterize in detail the hydrogen-bond network involving the formyl group of Chl *b*. The study revealed a variable degree of conformational freedom of the hydrogen bond networks among the WSCP variants, and an unexpected mixed presence of hydrogen-bonded and not hydrogen-bonded Chls *b* in the case of the L91P mutant of Lv WSCP. These findings helped to refine the description of the mechanisms underlying the different Chl *a*/*b* specificities of WSCP versions, highlighting the importance of the structural rigidity of the Chl binding site in the vicinity of the Chl *b* formyl group in granting a strong selectivity to binding sites.

## Introduction

**Figure 1:**
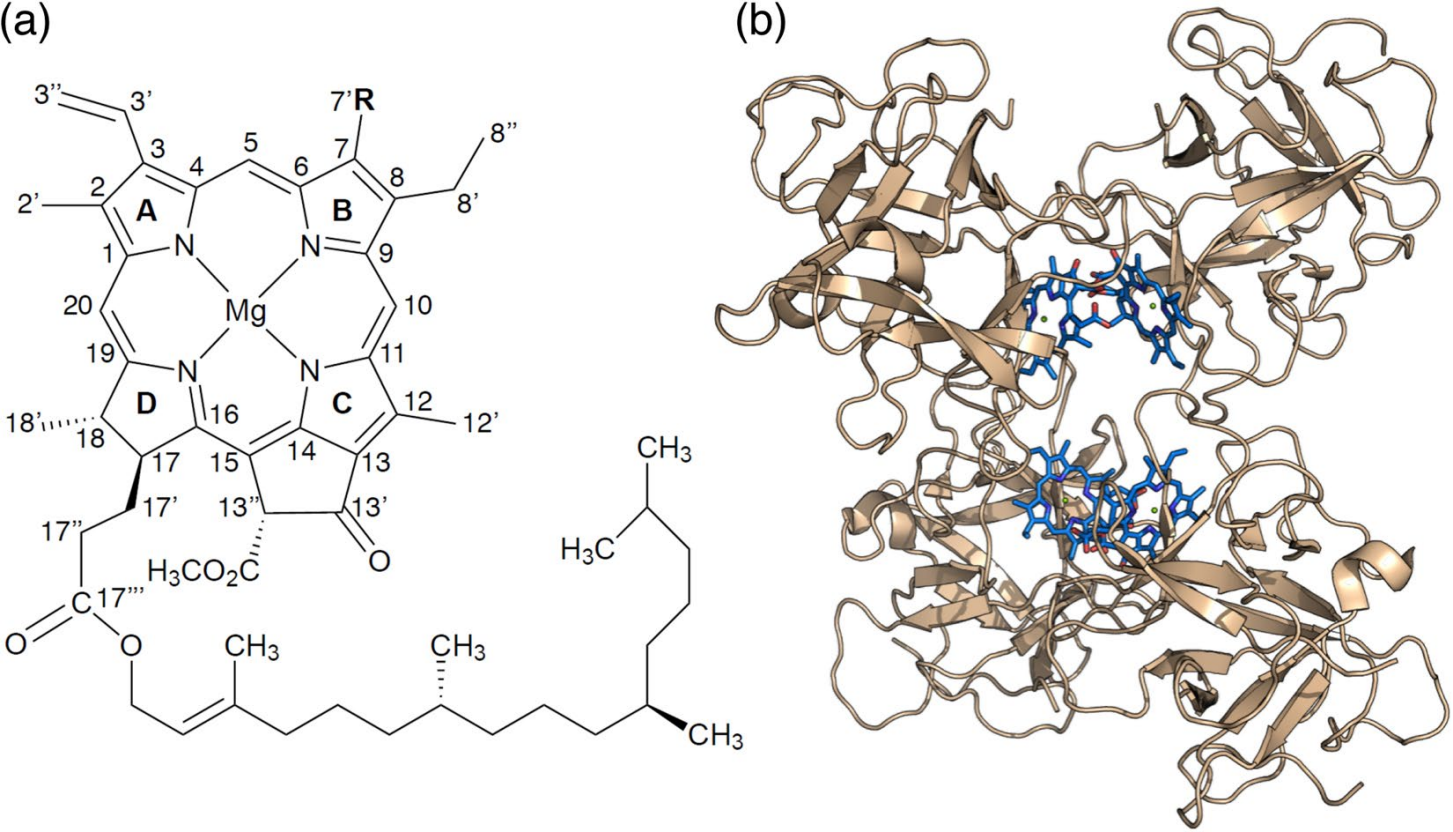
(**a**) Structure of Chl *a* (R = CH_3_) and *b* (R = CHO). (**b**) Crystallographic structure of Lv-*wt* WSCP^36^. Chls in sticks and the secondary structure in cartoon. Beige, polypeptide; blue, Chl *a*. The phytyl chains have been omitted for clarity.

Hydrogen (H)-bonds are fundamental short-range interactions in biological macromolecules^1^, especially in proteins. They are essential in giving rise to the multifarious structures and functions that this class of macromolecules displays in the whole kingdom of life. H-bonds stabilize the folding of proteins^2^, and contribute to both their catalytic mechanisms^3,4^ and the ligation of cofactors that they require to accomplish their physiological function^5^. This latter role is of paramount relevance for proteins involved in the photosynthetic process, as they need to bind numerous pigments, such as chlorophylls (Chls), in a precise three-dimensional arrangement to effectively harvest and utilize incoming light^6^. Numerous mutational studies have been performed on photosynthetic proteins, that helped to highlight the role of individual H-bonds in determining the spectroscopic properties of the bound pigments^7–14^. Beside modulating the properties of the bound Chls^7–10,15^, the presence of an appropriate H-bond donor in close proximity to the C-7^1^ atom of the Chls (see Fig. 1a) seems to be a strong predictor for Chl *b-*binding sites in plant proteins^16,17^.

In fact, this is the only part of the molecule bearing a group that distinguishes Chl *a* and *b*, as they display either a methyl or a formyl group in this position, respectively (Fig. 1a). The formation of an H-bond between the Chl *b* formyl group and its protein surrounding has been proposed to play an important part in the stabilization of the main light-harvesting complex in plants^18,19^. Chl *b* acts as an auxiliary light-harvesting pigment, thanks to its absorption in spectral regions where the absorption of Chl *a* is less intense^20–22^. Additionally, Chl *b* has been proposed to play an essential role in the regulation of the antenna size in higher plants^23–27^, an important mechanism in the acclimation to high light conditions^26,28^. This regulation is possible because Chl *b* is bound only by peripheral antenna components belonging to the LHCs superfamily and is required for their folding^19,29^.

Despite being recognised as a fundamental mechanism for the correct assembly and functionality of light-harvesting complexes, the molecular details of the H-bond-mediated ligand-protein recognition are still largely unknown. To shed light on the specific molecular interactions promoting the formation of these H-bonds, Water-Soluble Chlorophyll Proteins (WSCP) represents an ideal model system^17^. WSCPs are a class of non-photosynthetic Chl-binding proteins from plants^30^. These homo-tetrameric proteins^31,32^ are present only in the plant family of *Brassicaceae* and contain only one Chl per monomer^33–35^ in symmetrically related and structurally identical binding sites (Fig. 1b)^36,37^. Interestingly, different WSCPs are characterized by different relative affinities for Chl *a* or Chl *b*. While wild type (*wt*) WSCP from Virginia pepperweed (*Lepidium virginicum*; hereafter denoted as Lv-*wt*) is a Chl *b*-preferring protein^17,38^, this is not true for the *wt* WSCP from Cauliflower (*Brassica oleracea* var. *Botrytis*; hereafter denoted as Bo-*wt*) which exhibits no selectivity for either Chl *a* or Chl *b*^17,39^.

In a previous detailed mutational study, the comparison of these two WSCP variants allowed identifying a small 4 amino acid-long motif (-LCPS-) in a loop near ring B of the Chl as the source of the marked Chl *b-*selectivity displayed by Lv-*wt*^17^. The 2 amino acids-longer motif (-PVCNEL-) found in Bo-*wt* at the corresponding position does not lead to a preference for either Chl *a* or Chl *b*. From the inspection of the available crystallographic structures^36,37^, the observed differences in Chl selectivity were attributed to the possibility of forming an effective H-bond between the LCPS or PVCNEL motifs and the formyl group at the C-7^1^ position of Chl *b*^17^. In the same study^17^, it was found that the exchange of the L91 residue in Lv-*wt* by a proline converts the protein into a Chl *a*-preferring one. This single amino acid substitution remarkably increases the relative Chl *a*/*b* binding affinity by a factor of around 40. The Lv-L91P-Chl *b* crystal structure revealed that the nearest group potentially able to form an H-bond with the C-7^1^ carbonyl (the backbone nitrogen of C92) is too far away (4.9 Å) for a direct H-bond. Instead, a water molecule acts as a bridge giving rise to a H-bond network. The bulkier formyl group and the additional water molecule increase the steric hindrance in the binding pocket. This leads to a switch of P91 into its energetically unfavourable *exo*-conformation^40^, resulting in the lower selectivity of Lv-L91P for the binding of Chl *b*^17^.

These findings call for an in-depth structural characterization to unveil the molecular basis at the origin of the observed difference in Chl *a*/*b* affinity. To this end, we coupled a crystallographic approach, solving the still missing Chl *b*-reconstituted *wt* structures (Chl *b* Lv-*wt* and Chl *b* Bo-*wt*), to a Raman investigation focused on the carbonyl groups, with the aim to characterize the (possible) H-bond in which the carbonyl may be involved. The biggest advantage of this vibrational technique, which makes it complementary to crystallography, is the possibility to study the protein in solution at physiological temperatures. Its very high selectivity in complex media allows following variations of molecular interactions, such as H-bonds between Chl *b* and its surrounding, at experimental conditions not accessible to crystallography^41^.

## Results

### Structure determination of the Chl *b* Lv-*wt* and Chl *b* Bo-*wt*

Lv-*wt* was solved to 2.3 Å and Bo-*wt* to 2.5 Å (Table S1) with the molecular replacement method, using the structures of Chl *a-*binding WSCPs as models (2DRE^36^ and 5HPZ^37^ for Chl *b* Lv-*wt* and Chl *b* Bo-*wt*, respectively). Comparison of Chl *b* Lv-*wt* with Chl *a* Lv-*wt*^36^, as well as Chl *b* Bo-*wt* and Chl *a* Bo-*wt*^37^, reveals no difference in the quaternary structures, with the backbone traces almost coincident (see Fig. S1).

**Figure 2:**
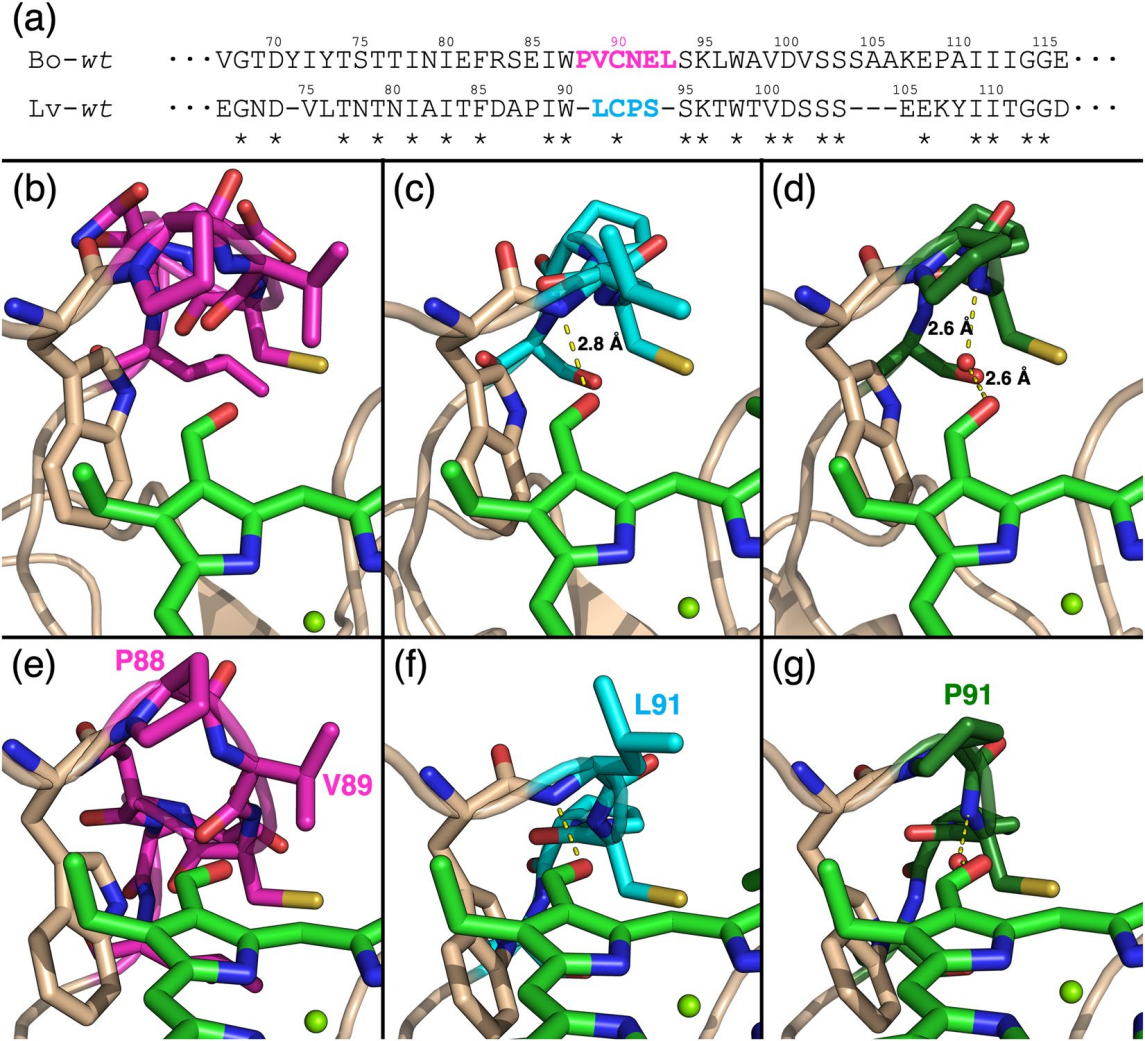
Interaction between the different Chl-selectivity motifs and bound Chls *b*. (**a**) Section of the polypeptide sequence alignment of the mature WSCP from *Brassica oleracea* var. *botrytis* (Bo) (uniProt: Q7GDB337^31^) and from *Lepidium virginicum* (Lv) (uniprot: O0479735^39^) in which the affinity motifs are found. ClustalW was used for the alignment^56^. Conserved amino acids are marked by an asterisk. The affinity motifs in Chl-binding loop structures are colored in magenta (PVCNEL, in Bo-*wt*) and in cyan (LCPS, in Lv-*wt*). (**b**,**e**) crystallographic structure of Bo-*wt* (PDB: 6S2Z). (**c**,**f**) Crystallographic structure of Lv-*wt* (PDB: 6S2Y). (**d**,**g**) crystallographic structure of Lv-L91P (PDB: 6GIX^17^). The Chls *b* are shown in light green, the Chl-selectivity motifs in magenta (PVCNEL), cyan (LCPS), and dark green (PCPS), other residues in beige. The oxygen atom of the water molecule in Lv-PCPS is shown as a red sphere. The distances between the C-7^1^ carbonyl group and the nearest H-bond donors are indicated by yellow dashed lines.

The analysis of the surroundings of the bound Chls *b* (focused on their B pyrrole in Fig. 2) confirmed the assumptions previously made on the basis of the available Chl *a* reconstituted WSCP structures^17^. Thus, for Lv-*wt*, we can confirm that the peptidic nitrogen of L91, part of the LCPS motif (cyan in Fig. 2c,f), is positioned at the right distance and orientation from the formyl oxygen of Chl *b* to provide a H-bond.

**Figure 3:**
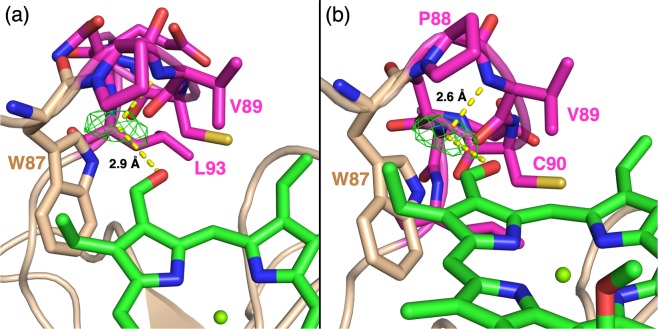
Electron density maps of Chl *b-*reconstituted Bo-*wt*. Omit electron density map *F*_*o*_ − *F*_*c*_, contoured at 3.0 σ, is shown in green when positive in all the panels. The distances of the center of the electron density and its nearest H-bond partners are indicated by yellow dashed lines. The phytyl have been omitted for clarity. The coloring of the residues is consistent with Fig. 2.

The PVCNEL motif (magenta in Fig. 2b,e) of Bo-*wt* possesses two more amino acids and thus a 50% increased length compared to the corresponding LCPS sequence of Lv-*wt*. This leads to a distortion of the loop in Bo-*wt*. The increased length of the PVCNEL motif forces the potential H-bond partner (backbone nitrogen of V89) away from the C-7^1^ carbonyl, resulting in a distance between the backbone nitrogen of V89 and the formyl oxygen of pyrrole ring B of 4.0 Å. Consequently, direct interaction is prevented. A faint electron density has been identified at 2.6 Å from the backbone nitrogen of V89 and 2.9 Å from the C-7^1^ carbonyl (Fig. 3), that would lead to the formation of H-bonds of moderate strength^42^ if assigned to a water molecule. However, due to its very weak appearance, we refrained from an assignation in the crystallographic structure (Fig. 2b,e).

### Raman of Chl *b* reconstituted Lv-*wt*, Lv-L91P, and Chl *b* Bo-*wt*

**Figure 4:**
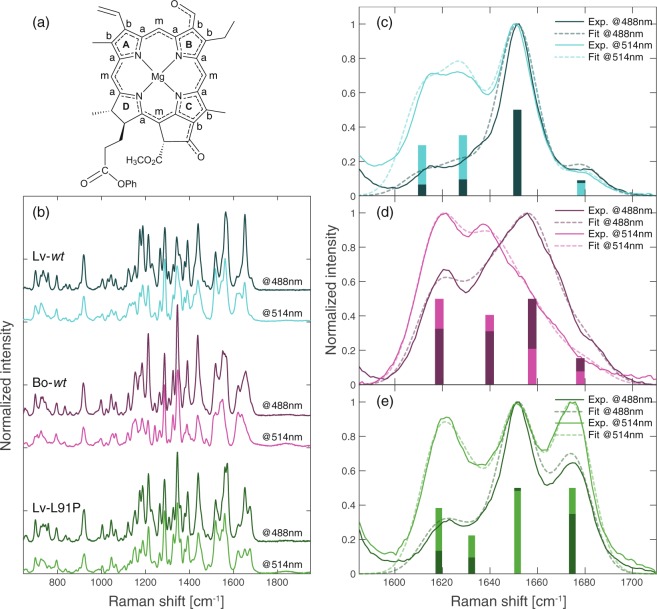
(**a**) Molecular structure of Chl *b* where π-bond delocalization is highlighted. (**b**) Full Raman spectra of Chl *b* in Lv-*wt* (top), Bo-*wt* (middle), and Lv-L91P (bottom) (buffer solutions). Two distinct exciting conditions are considered, 488.0 nm (dark colors) and 514.5 nm (light colors). Global fitting analysis was performed on the two spectra recorded for Lv-*wt* (**c**), Bo-*wt* (**d**), and Lv-L91P (**e**) within 1585–1710 cm^−1^ frequency range. The model functions built in the fitting routine are multi-Gauss functions reported as dotted lines. The center and relative amplitude of the Gaussian components are described with bars bearing the same color code of Raman spectra.

A perfect probe to investigate the C-7^1^ carbonyl oxygen and its interactions with the surroundings, particularly through H-bonds, is the frequency of the C=O stretching. To further investigate the H-bonds involving the C-7^1^ carbonyl oxygen, a Raman characterization has been carried out on phosphate buffer solutions of the Chl *b* reconstituted WSCP variants discussed in this study (Bo-*wt*, Lv-*wt*, and Lv-L91P) at two distinct exciting conditions (488.0 and 514.5 nm). The excitation at 514.5 nm is essentially non-resonant, whereas 488.0 nm can be considered nearly resonant with the Soret bands of Chl *b*. The comparison of resonant and non-resonant Raman spectra provides information on the modes more strongly coupled with the resonant electronic transition and can help to disentangle the individual contribution of single components in complex spectra. The attention has been focused in particular on the 1600–1700 cm^−1^ region, as shown in Fig. 4, where the contribution of the C=O stretching modes is expected^43,44^.

In Chl *b*, four carbonyl groups are present: (i) the C-7^1^ formyl group, (ii) C-13^1^ keto group, (iii) C-15^2^ and (iv) C-17^3^ esteric carbonyl. However, the latter three do not contribute significantly to Raman spectra of Chls, due to a low degree of conjugation with the chlorin plane^43^. The frequency of the C=O stretching is determined by the strength of the C=O bond, which, in turns, is strongly affected by the possible involvement of the oxygen in H-bonds. In particular, H-bonds reduce the C=O bond strengths, and consequently their stretching frequency. Indeed, the free formyl stretching mode was determined for Chl *b* at 1670 cm^−1^, whereas the H-bonded formyl stretching mode was found^44^ to be red shifted at 1647 cm^−1^. Therefore, the position and the shape of the C=O stretching mode signal in the Raman spectra are expected to be good reporter of the presence of H-bonds formation.

The Raman spectra of the three samples in the 1600–1700 cm^−1^ region of interest, both in resonant and non-resonant conditions, are reported in Fig. 4c,e. The behaviour across the samples is very different, both in terms of peak position and width.

A complication in the analysis of the spectra comes from the contribution of C=C stretching modes falling in the same spectral window. Indeed, according to Lutz^44^, C_a_C_m_ partial double bond stretching modes (where C_a_ are pyrrolic carbons and C_m_ are methine carbons, see Fig. 4a) contribute to the signal in the 1600–1630 cm^−1^ region. While a detailed interpretation of these modes goes beyond the goals of this paper, their partial overlap with the C=O stretching modes under investigation requires anyway to take them into consideration in the spectra analysis. To achieve a precise determination of the spectral position of the different signals and verify possible shifts due to H-bond formation, the Raman spectra recorded in resonant and non-resonant conditions have been analysed by means of a global fitting procedure. Specifically, a global analysis allows to fit both the spectra recorded at different excitation wavelengths with the same spectral features (peak positions) but with different relative amplitudes ad widths. This approach allows imposing an increased number of experimental constrains to the fit, and therefore it is expected to provide more robust results. The fitting function is built as a sum of a tuneable number of Gaussians. For each sample, we found that the minimal number of components that satisfactorily fits the Raman spectra is four. The fitted parameters are reported in Table 1, and the fitting functions in Fig. 4c,e as dotted lines. Both in Lv-*wt* and in Lv-L91P, two contributions in the CC partial double bond stretching modes region have been identified, while a single component was sufficient to fit the same region in the Bo-*wt* sample.

**Table 1:** Global fitting parameters of the Raman spectra.

	E_1_ [cm^−1^]	E_2_ [cm^−1^]	E_3_ [cm^−1^]	E_4_ [cm^−1^]	FWHM [cm^−1^]
Lv-*wt*	1612	1629	1652	1678	19
Bo-*wt*	1619	1640	1658	1678	21
Lv-L91P	1618	1632	1652	1675	19

Centers (E_n_) and full width at half maximum (FWHM) of the gaussian components.

In Lv-*wt*, as described above, we found a main peak centred at 1652 cm^−1^ and a minor feature at 1678 cm^−1^. In Bo-*wt* instead, three distinct components are needed to fit the Raman spectrum: two components at 1640 and 1658 cm^−1^, and a weak signal at 1678 (as already seen in Lv-*wt*). Lv-L91P shows two almost equally contributing peaks, at 1652 and 1675 cm^−1^.

It is interesting to note that in the case of Bo-*wt*, it was necessary to adopt slightly wider components (+11%) in order to fit the spectra in comparison to the other two complexes, due to the broadness of the recorded signals.

Taking into considerations the experimental and fitting results together with the assignments from previous works^43,44^, we can attribute the intense peak at 1652 cm^−1^ that is present both in Lv-*wt* and Lv-L91P to the stretching of the H-bonded C-7^1^ formyl group.

In Lv-*wt*, this component is predominant, and this could be the consequence of a direct H-bond between the formyl group and a peptidic nitrogen, as this is expected to lead to a rigid conformation, with a well-defined distance between the H-bond donor and the acceptor.

In the case of Lv-L91P, the Raman spectrum reveals a further strong signal at 1675 cm^−1^ attributable to free formyl stretching. This is a novel finding, that could not be captured by the crystallographic structure, in which all the formyls appeared to be involved in H-bonds with a water molecule characterized by an occupancy of 1^17^. The two peaks show a comparable intensity, suggesting that in solution the Chl binding pockets are characterized by a heterogenous mixture of configurations with and without the H-bonded water molecule.

Bo-*wt* presents an intense peak at 1658 cm^−1^, at a frequency only slightly higher than the one observed in Lv-*wt*. This frequency is compatible with an H-bonded formyl group^44^ but the higher frequency suggest that the hydrogen bond is weaker in this protein, a finding that is in good agreement with the higher distance suggested from the crystallographic structure (Fig. 3a,c). As demonstrated by the global fit, Bo-*wt* presents broader peaks in the formyl stretching region, probably due to a broader distribution of the distance between the formyl oxygen and its H-bond partner that, in analogy to Lv-L91P, could be a water molecule H-bonded to the peptidic nitrogen of V89 (see Fig. 3). The broadness of this Raman signal may explain the faint and elongated electron density that we found in the omit map of the Bo-*wt* structure (Fig. 3), as it could be originated by a less strictly defined position of this water molecule in the binding site, with a consequent broad distribution of C-7^1^=O stretching modes in Bo-*wt*.

## Discussion

Our combined Raman and X-ray investigation of Chl *b-*reconstituted WSCP variants confirmed our previous hypothesis^17^ that in Lv-*wt*, the protein backbone of the LCPS motif provides a H-bond donor at an appropriate distance from the C-7^1^ group (Fig. 2c), leading to the formation of a stable and well defined H-bond, as judged from the narrow distribution of the C-7^1^=O stretching peak (Fig. 4c) in Raman spectra. This interaction is expected not only to contribute to the stabilization of the complex^45^, but also to be the origin of the marked Chl *b* selectivity displayed by this WSCP variant^17^. This is analogous to the case of the light-harvesting 2 complex (LH2) of purple phototrophic bacterium *Rhodobacter sphaeroides*, in which an arginine residue has been proven to play a crucial role in binding specificity of the complex, by providing a hydrogen-bond to the C-3^1^ acetyl group of the native pigment (bacteriochlorophyll *a*)^46^. In this context, a comparison with the LH2 complex of another purple phototrophic bacterium, *Phaeospirillum molischianum*, is instructive. Here, the C-3^1^ acetyl group of BChl *a* interacts with the protein via a threonine, providing one uncharged polar hydrogen bond, whereas in *P*. *sphaeroides*, the corresponding interaction occurs via an arginine, providing two charged hydrogen bonds. As a consequence, this interaction has been proposed to contribute more significantly to the stabilization of pigment binding in the LH2 of *P*. *sphaeroides* compared to that of *P*. *molischianum*^47^.

In Bo-*wt*, the increased distance of the first suitable H-bond donor in the backbone of the PVCNEL motif seems to be compensated by an alternative H-bond donor at a suitable distance from the formyl group. Its assignment to a water molecule is based on the small available space inside the binding pocket and to the faintness of its electron density in the crystallographic structure. This water molecule appears to provide an H-bond with a broader distribution of strengths, as indicated by its lack in the crystallographic structure and the broader distribution of the C-7^1^=O stretching peak (Fig. 4d) if compared to the corresponding peak in Lv-*wt* (Fig. 4c). Therefore, the position of the water molecule inside the Chl binding pocket appears to be not rigidly defined, both in the crystal and in solution.

Lv-L91P has already shown to provide an H-Bond to the C-7^1^ formyl group of Chl *b via* the ligation in the Chl binding site of a water molecule (Fig. 2d)^17^, similarly to Bo-*wt*. Even if the crystallographic structure shows a clear electron density in all the binding sites of the complex, giving rise to an occupancy of 1 for the water molecules in H-bond, the Raman spectra reveal two sharp and well resolved signals of comparable intensities attributable to H-bonded and free formyl groups (Fig. 4e). This evidence points to the coexistence of complexes in solution with and without a water molecule in the binding site. The two contributions appear to be quite narrow, suggesting that in Lv-L91P the water molecule, when present, has a position in the binding site more rigidly determined than in Bo-*wt*. This situation is more similar to the H-bonding action of the peptidic nitrogen provided by the backbone of V89 in Lv-*wt*. This bi-modal distribution of binding sites was not found in the crystal, that appears to be composed only of complexes where a water molecule is involved in an H-bond with each Chl *b*, suggesting that these complexes are favoured in the crystallization process.

Water-mediated H-bond ligation of C-7^1^ formyl is a structural feature that can be found also in photosynthetic complexes, where it plays an important role in the Chl *b* selectivity of certain binding sites^17^. In LHCII, the major light-harvesting complex of higher plants, Chl *b*606 shows this kind of interaction. In this case the water molecule acts also as the fifth ligand of the magnesium of another Chl (*b*607)^16^, hence it can be expected to be present in all the complexes of the LHCII population, as its lack would mean the absence of the bound Chl *b*. Accordingly, it has been observed that the mutation of the residue ligating the magnesium Chl *b*606 (Q131A) leads to the loss of both the Chls *b*^48^.

**Figure 5:**
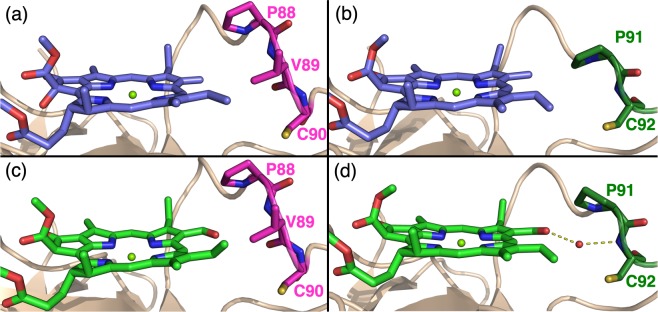
Sections from four WSCP crystal structures highlighting the conformation of a proline in close distance to pyrrole ring B of bound Chl *a* or *b*. Carbons are colored blue (Chl *a*), light green (Chl *b*), magenta (Bo-*wt*) or dark green (Lv-L91P). Oxygen atoms are colored red, nitrogen blue, sulfur yellow and magnesium light green. A water molecule is displayed as a red sphere. The secondary structure is shown in beige cartoons. H-bonds are shown as yellow dashed lines. Phytyls are omitted for clarity. (**a**) *Endo*-conformation of P88 in BoWSCP-Chl *a* structure (PDB: 5HPZ^37^). (**b**) *Endo*-conformation of P91 in Lv-*wt*-Chl *a* structure (PDB: 6GIW^17^). (**c**) *Endo*-conformation of P88 in Bo-*wt* Chl *b* structure (PDB: 6S2Z). (**d**) *Exo*-conformation of P91 in Lv-L91P-Chl *b* structure (PDB: 6GIX^17^).

Besides the presence of a suitable H-bond donor for the C-7^1^ carbonyl, we found that in WSCP also the steric interaction with other amino acids in the close proximity is a relevant factor in dictating the selectivity towards Chl *a* or *b*. In the Lv-L91P mutant, the binding of Chl *b* and a water molecule was shown to cause a steric clash with the P91 residue, resulting in its switch to the unfavourable *exo-*conformation (Fig. 5d) from the thermodynamically favoured^40^ *endo-*conformation adopted when Chl *a* was bound (Fig. 5b). Bo-*wt* also has a proline residue in the binding pocket, close to ring B (Fig. 5a,c), but the residue conformation is not affected by the binding of Chl *b* and a water molecule, as it retains its *endo*-conformation (Fig. 5c). This is in accordance with the observation that Bo-*wt* does not preferentially bind Chl *a*, contrarily to Lv-L91P^17^.

The remarkable Chl *b* selectivity of Lv-*wt*^17,38^ can be attributed to the presence of an appropriate H-bond partner pre-oriented at the correct distance and orientation from the ring B of the bound Chl, as evident from the comparison of the two structures, to give rise to a strong interaction. The [L/P]CPS motif, shared by Lv-*wt* and Lv-L91P, seems to lead to similarly narrow Raman signals despite the difference in the H-bond partner between the two variants originated from the substitution of the residue in position 91. So, the narrower distribution of H-bond strengths seems to be attributable more to the rigidity of the binding site than to the identity of the H-bond partner. Thus, the shorter 4 amino acid length of the [L/P]CPS motif provides a more rigid binding site, whereas the longer PVCNEL determines a higher heterogeneity in the surrounding of C-7^1^ in solution, which can be attributed to a higher flexibility. This nicely fits with the capability of the binding sites to accommodate a water molecule upon binding Chl *b*: in Bo-*wt* the water molecule is accommodated in the totality of the complexes, suggesting an easier fit, whereas in Lv-L91P there is a pool of binding sites in which the water molecule is not present. This can be attributed to a more rigid binding site, as the binding of the water molecule in Lv-L91P requires an unfavourable switch of the confirmation of P91^17^ (see Fig. 5). All these considerations concur in the explanation of the lack of selectivity^17,39^ for the more flexible Bo-*wt* binding site, and the preference for Chl *a* shown by the more rigid Lv-L91P binding site^17^.

## Conclusions

The present study highlights the complementarity of X-ray and Raman for structural investigations of H-bonds mediating chromophore-protein interactions. On one hand, the crystallographic characterization provides rich information regarding the whole structure, allowing to investigate in great detail the chromophore binding site. On the other hand, Raman spectra, that selectively focus on a specific chemical group and its close surroundings, can be conducted under conditions closer to the physiological ones. This advantage of Raman spectroscopy allowed us to provide additional information, which were not accessible from the investigation of the crystal alone in the case of Lv-L91P variant. In fact, our study highlighted the possibility for crystals of protein-pigment complexes to exhibit only one of the two configurations adopted in solution. Additionally, the Raman characterization proved to be fundamental also in the case also of Bo-*wt*, as it allowed to reveal the presence of an H-bond partner for the bound Chl *b*, not discernible in the crystallographic structure due to the flexibility of the binding site.

In the light of the relevance of these kind of interactions in cofactor selection and binding in proteins, we would like to stress the need of pairing complementary techniques to X-ray crystallography when structure-function relationships are investigated. The combined approach of X-ray and Raman presented in this study led to an update of our interpretative model of the Chl *a*/*b* selectivity in WSCPs, highlighting that the rigidity of the surrounding of Chl’s ring B in Lv leads to selectivity either towards Chl *b* when a suitable H-bond partner is present (Lv-*wt*), or towards Chl *a* when a H-bond partner, such as water, has to be incorporated in a sterically hindered binding site (Lv-L91P). Accordingly, when the surrounding of ring B is less hindered, like in Bo-*wt*, there is little or no selectivity.

Structural rigidity in the surrounding of ring B of Chls as the prerequisite of Chl *a*/*b* selectivity is likely to extend to other Chl-binding proteins. This may be of high relevance in all those cases in which Chl *b* is functionally important, as in the case of energy funnelling in light harvesting complexes.

## Material and Methods

### Sample preparation

Chl *b* was extracted and purified from pea plants (*Pisum sativum*) according to Booth *et al*.^49^. Protein overexpression in *E*. *coli* and following purification have been performed as previously reported^50^. The purified WSCP apoproteins were reconstituted with Chl *b*, as previously described^17^. Prior to further investigations, the samples (dissolved in 20 mM sodium phosphate; pH 7.8) were analysed via absorption and circular dichroism spectroscopy in order to verify their correct assembly, obtaining spectra comparable to those expected^17,34,50^.

### Crystal structure determination and refinement of Lv-*wt* Chl *b* and Bo-*wt* Chl *b*

Crystallization of Lv-*wt* Chl *b* was performed by hanging-drop vapour diffusion adapted from conditions previously reported^17,36^: 6.0 mg/ml of protein in a 0.1 M Na/K phosphate buffer (pH 6.0) with 3.0 M (NH_4_)_2_SO_4_ and 4.7% sucrose. Green rhombic crystals appeared after one week and grew to their final size of 0.3 × 0.3 × 0.3 mm within three weeks. Crystallization of Bo-*wt* Chl *b* was performed by hanging-drop vapour diffusion with conditions adapted from Bednarczyk *et al*.^37^: 5.5 mg/mL of protein in a 0.1 M (NH_4_)H_2_PO_4_ (pH 4.5) solution containing 10% PEG 3350. Green oblong crystals appeared after one week and grew to their final size of 0.3 × 0.1 × 0.1 mm within two weeks.

Crystals were flash cooled and measured in the gas stream of a cryostream system (Oxford Cryosystems), with a nitrogen gas temperature of 100 K. Due to the sucrose and ammonium sulfate content of the mother liquor, no additional cryoprotectant was necessary for Lv-*wt* Chl *b*. In the case of Bo-*wt* Chl *b*, the soaking of a crystal in a buffer solution (0.1 M (NH_4_)H_2_PO_4_ pH 4.5) containing 30% ethylene glycol prevented the formation of ice rings.

Data were collected using a Microstar rotating anode (Bruker AXS) and a ‘mar345’ image plate detector (MARresearch). For Lv-*wt* Chl *b* data were collected for 119° with an increment of 1.0° and a crystal to detector distance of 150 mm. For Bo-*wt* Chl *b* data were collected for 194° with an increment of 1.0° and a crystal to detector distance of 150 mm. Data were collected up to a resolution of 2.3 Å for Lv-*wt* and 2.5 Å for Bo-*wt*, and processed with the XDS program package (Version: Mar 15, 2019^51^). Crystal parameters, data collection parameters and refinement statistics are given in Table S1. The structure solution was obtained by molecular replacement with the corresponding Chl *a* containing complex as starting model (2DRE^36^ and 5HPZ^37^ for Lv-*wt* Chl *b* and Bo-*wt* Chl *b*, respectively), using the program PHASER implemented in the CCP4 suite^52,53^. The structure was refined using the program Coot/REFMAC^53,54^. The final models and structure factors were deposited in the PDB databank with accession code 6S2Y (Lv-*wt* Chl *b*) and 6S2Z (Bo-*wt* Chl *b*). Molecular graphics were produced using PyMOL Molecular Graphics System (DeLano Scientific).

### Raman spectroscopy

Raman spectra have been performed directly on phosphate buffer solutions (20 mM sodium phosphate; pH 7.8) of WSCP with a home-built micro-Raman system, previously described^55^. Briefly, it is based on a Triax-320 ISA spectrograph, equipped with a holographic 1800 g/mm grating and a CCD detector (Spectrum One ISA Instruments). The excitation sources were a Spectra Physics Ar^+^ laser (Stabilite 2017) operating at 488.0 nm and 514.5 nm for nearly resonant and non-resonant conditions, respectively. Appropriate edge filters were used to reduce the stray-light level. An Olympus BX40 optical microscope equipped with a long working distance 20x/0.40 objective was optically coupled to the spectrograph. The Raman spectra were recorded on WSCP solutions between 645 and 2000 cm^−1^ and with an instrumental resolution of about 2 cm^−1^. To avoid optical damage the exciting radiation was maintained between 4 and 6 mW.

The value of the Raman shift of the signals appearing in the nearly resonant and non-resonant spectra are reported in Table S2.

## Supplementary information

Supplementary Information

## Data Availability

The authors declare that the data supporting the findings of this study are available from the corresponding authors upon request. Crystal structures determined in this study have been deposited in the Protein Data Bank (http://www.rcsb.org), with accession code 6S2Y (Lv-*wt* Chl *b*) and 6S2Z (Bo-*wt* Chl *b*).
