# Supplementary material for: How water-mediated hydrogen bonds affect chlorophyll a/b selectivity in Water-Soluble Chlorophyll Protein
Source: Sci Rep. 2019 Dec 3;9:18255. doi: 10.1038/s41598-019-54520-4 (PMC6890793; doi:10.1038/s41598-019-54520-4)
Supplement: Supplementary file 1 — Supplementary Information [file 41598_2019_54520_MOESM1_ESM.pdf]

SUPPORTING INFORMATION

How water-mediated hydrogen bonds affect chlorophyll *a*/*b* selectivity in Water-Soluble Chlorophyll Protein

Alessandro Agostini[1,2,*], Elena Meneghin[1], Lucas Gewehr[2], Danilo Pedron[1], Daniel M. Palm[2], Donatella Carbonera[1], Harald Paulsen[2], Elmar Jaenicke[3], and Elisabetta Collini[1,*]

[1]Department of Chemical Sciences, University of Padova, via Marzolo 1, 35131, Padova, Italy.

[2]Institute of Molecular Physiology, Johannes Gutenberg-University, Johannes-von-Müller-Weg 6, 55128 Mainz, Germany

[3]Institute of Molecular Physiology, Johannes Gutenberg-University, Jakob-Welder-Weg 26, 55128 Mainz, Germany

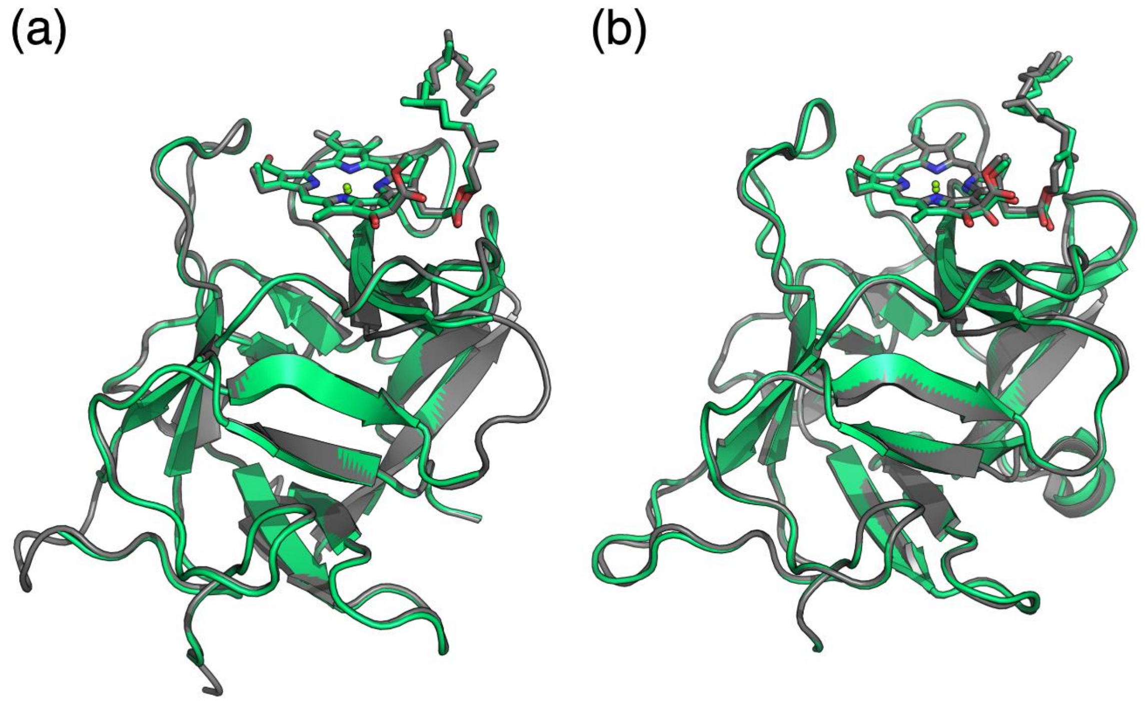

Figure S1: Comparison of the aligned crystallographic structures of **A**) Lv-*wt* reconstituted with Chl *a* (grey; PDB: 2DRE[1]) and Chl *b* (green; PDB: 6S2Y), and **B)** Bo-*wt* reconstituted with Chl *a* (grey; PDB: 5HPZ[2]) and Chl *b* (green; PDB: 6S2Z). The comparison is focused on a monomer, with the secondary structure shown in cartoons and the Chls in sticks.

Table S1: Data collection and refinement statistics (Molecular Replacement).

	Lv-*wt* Chl *b* (PDB: 6S2Y)	Bo-*wt* Chl *b* (PDB: 6S2Z)
**Data collection**		
Space group	P 2_1 2_1 2_1	C 2 2 2
Cell dimensions		
a, *b*, *c* (Å)	73.31, 82.90, 122.22	86.36, 118.33, 38.55
α, β, γ (°)	90.0, 90.0, 90.0	90.0, 90.0, 90.0
Resolution (Å)	19.63 – 2.30 (2.36 - 2.30)*	19.72 – 2.50 (2.57 - 2.50)
R_{merge}	0.078 (0.461)	0.180 (0.717)
$CC_{1/2}$	0.997 (0.797)	0.995 (0.879)
$I / \sigma I$	12.31 (3.03)	15.82 (3.78)
Completeness (%)	98.7 (99.8)	99.5 (99.6)
Redundancy	4.62 (4.76)	7.51 (7.51)
**Refinement**		
Resolution (Å)	19.63 - 2.30	19.72 - 2.50
No. reflections	31655	6790
R_{work} / R_{free}	0.243 /0.276	0.217 /0.284
No. atoms		
Protein	4914	1323
Ligand/ion	264	66
Water	130	22
B-factors (Å^2)		
Protein	44.07	25.37
Ligand / ion	31.25	17.52
Water	35.28	19.60
R.m.s. deviations		
Bond lengths (Å)	0.008	0.007
Bond angles (°)	2.390	2.354
Ramachandran plot (%)		
Most favored	94.03	94.80
Allowed	5.31	4.60
Disallowed	0.66	0.60

*Values in parentheses are for highest-resolution shell.

Table S2: Raman shift [cm^{-1}] of vibrational modes recorded on phosphate buffer solutions of Chl *b* reconstituted WSCP variants at two exciting conditions (488.0 and 514.5 nm).

**Lv-*wt***		**Bo-*wt***		**Lv-L91P**	
**488.0 nm**	**514.5 nm**	**488.0 nm**	**514.5 nm**	**488.0 nm**	**514.5 nm**
652	650	652	652	653	
		654	656	656	655
662	661	662	661	659	661
672	671				
700	700	699	699	700	699
			708		709
725	726	726	726	725	725
735		733	737	735	734
		745	742	745	741
757	758	757		759	760
		765	764		764
797	797	797	794	796	795
828	826		832	830	
838	837	834		838	837
855	854	856	856	852	851
883	884	887	885	886	885
904	902			905	902
919	919	918	918	921	922
			951		
	983	981	982	985	988
1004	1003	998	997	1003	1004
	1029	1022	1023	1025	1026
1044	1045	1044	1050	1044	1045
1064	1064	1065	1064	1064	1064
1088		1089	1092	1088	1091
	1098				
1106	1104	1106	1104	1106	1104
1122	1128	1122	1120	1121	1122
					1131
	1141				
1152	1152	1153	1154	1153	1153
1174	1174	1174	1171	1174	1174
1188	1187	1185	1184	1187	1188
1213	1213	1213	1211	1213	1214
1229				1228	
	1242	1242	1242		1240
1266	1266	1264	1265	1265	1265
1288	1287	1286	1286	1286	1287
1305	1305	1306	1305	1307	1307
1326	1326	1326	1325	1326	1327
1342	1343	1346	1347	1346	1345
1356				1360	1360
1378	1378	1380	1379	1380	1378
1391	1389	1390	1388	1391	1393
			1400		
			1413		1414
	1424				
1439	1438	1438	1438	1439	1439
1468				1465	
1482	1484	1478	1475	1483	1486
1519	1518	1519	1518	1518	1519
	1546	1551	1550		1547
1565	1562	1560		1561	1560
		1566		1571	
1615	1616	1620	1622	1623	1622
	1626				
			1637		
1652	1650	1656		1652	1651
1679	1679	1682		1674	1676

References

1. Horigome, D. *et al.* Structural mechanism and photoprotective function of water-soluble chlorophyll-binding protein. *J. Biol. Chem.* **282**, 6525–6531 (2007).

2. Bednarczyk, D. *et al.* Chlorophyll Fine Tuning of Chlorophyll Spectra by Protein-Induced Ring Deformation. *Angew. Chemie Int. Ed.* **55**, 1–6 (2016).
